# Yacon (*Smallanthus sonchifolius* Poepp. & Endl.) as a Novel Source of Health Promoting Compounds: Antioxidant Activity, Phytochemicals and Sugar Content in Flesh, Peel, and Whole Tubers of Seven Cultivars

**DOI:** 10.3390/molecules23020278

**Published:** 2018-01-29

**Authors:** Forough Khajehei, Nikolaus Merkt, Wilhelm Claupein, Simone Graeff-Hoenninger

**Affiliations:** 1 Department of Agronomy, Institute of Crop Science, University of Hohenheim, Fruwirthstr 23, 70599 Stuttgart, Germany; claupein@uni-hohenheim.de (W.C.); simone.graeff@uni-hohenheim.de (S.G.-H.); 2 Department of Quality of Plant Products, Institute of Crop Science, University of Hohenheim, Emil-Wolff-Strasse 23, 70599 Stuttgart, Germany; merkt@uni-hohenheim.de

**Keywords:** yacon, *Smallanthus sonchifolius* Poepp. and Endl., sugar, total phenolic content, total flavonoid content, ABTS radical scavenging activity, DPPH radical scavenging activity, Ferric reducing antioxidant power

## Abstract

The aim of this study was to evaluate the quality characteristics of seven yacon (*Smallanthus sonchifolius* Poepp. and Endl.) cultivars (Cajamarca, Cusco, Early White, Late Red, Morado, New Zealand and Quinault) cultivated in the southwest of Germany. The following phyto/chemical traits were investigated in different yacon tuber parts (flesh, peel, and whole tubers): total dry matter, sugar content (fructose, glucose, and sucrose content), total phenolic content (TPC), total flavonoid content (TFC), 2,20-azino-bis(3-ethylbenzothiazoline-6-sulfonic acid) (ABTS) radical scavenging activity, 2,2-diphenyl-1-picrylhydrazyl (DPPH) radical scavenging activity, and Ferric reducing antioxidant power (FRAP). The results indicated a significant interaction between cultivar and tuber part on all of the examined traits (*p* < 0.0001). Of flesh and whole tuber, cv. Late Red, cv. Morado, and cv. Cajamarca had the highest TPC, TFC, DPPH radical scavenging activity, and FRAP. They also had relatively higher total sugar content. Cv. New Zealand had the lowest amount of sugars, TPC, TFC, DPPH radical scavenging activity, and FRAP, but the highest ABTS radical scavenging activity content in its flesh and whole tuber. Moreover, the results indicated that the peel of yacon tubers contained considerably high amounts of phytochemicals while possessing low sugar contents. Overall, this study provides a broad insight into the phyto/chemical content of yacon tubers from different cultivars, which can be used for further breeding programs, and the selection of proper cultivars for specific food product development.

## 1. Introduction

Consumption of fruits and vegetables is recommended as part of the human diet not only as a source of energy, but also as a source of health promoting compounds. Epidemiological researchers showed a favorable relationship between the consumption of fruits and vegetables and a reduction in risk of diseases such as cancer, cardiovascular diseases, etc. [1,2]. Phenolic compounds are secondary metabolites of plants and one of the most important groups of bioactive constitutes of fruits and vegetables due to their antioxidant activity [3,4]. Therefore, due to the phenolic profile of plant foods, their mechanism of action against certain diseases, health enhancing effects, safety, and potential in food products, plant-based nutraceuticals and pharmaceuticals are of interest and have been extensively investigated by researchers [5,6].

Yacon (*Smallanthus sonchifolius* Poepp. and Endl.) is a root crop native to the Andean region, but has also been cultivated in other parts of the world for example in Brazil, Czech Republic, Ecuador, Germany, Japan, and New Zealand [7]. Yacon tubers are crunchy and juicy with a relatively sweet taste and are traditionally consumed as fresh fruit [7]. 70–80% of the total dry matter content of yacon tubers consists of saccharides. They contain fructose, glucose, and sucrose as sugars while fructooligosaccharides (FOS) serve as their dominant saccharide [8,9,10]. FOS are prebiotic non-digestible carbohydrates, therefore yacon tubers have gained attention due to their potential not only as a part of a diet for those who are suffering from digestive disorders such as diabetes and obesity, but also as a health promoting food for dieters [11,12]. In the recent decade, several investigations have evaluated the amount of FOS in fresh yacon tubers or processed yacon products as well as their health benefits [11,13,14,15,16]. Besides having health promoting carbohydrates, yacon tubers contain bioactive compounds (e.g., phenolic compounds and antioxidants); accordingly, yacon is considered as a multifunctional food [7]. The total phenolic content (TPC) and antioxidant capacity of flesh of thirty-five accessions of yacon tubers, which were grown under Peruvian environmental conditions, have been investigated in a study of Campos et al. (2012) [17]. Their results showed that the TPC in the flesh of yacon tubers varied within a wide range of 7.9 ± 0.8 to 30.8 ± 0.1 (mg chlorogenic acid equivalent g^−1^ DW) and their antioxidant capacity ranged between 23.3 ± 2.5 and 136.0 ± 6.1 (µmol trolox equivalent g^−1^ DW) according to ABTS radical scavenging activity [17]. The average amount of TPC, DPPH radical scavenging activity, ABTS radical scavenging activity, and Ferric reducing antioxidant power (FRAP) of yacon flesh provided from three regional markets in Peru were 93.2 (mg gallic acid equivalent g^−1^ DW), 56.6 ± 0.4 (µmol trolox equivalent g^−1^ DW), 61.6 ± 0.8 (µmol trolox equivalent g^−1^ DW), and 134.0 ± 7.2 (µmol trolox equivalent g^−1^ DW), respectivly [18]. Sousa et al. (2015) reported the total antioxidant capacity of sterilized flour of yacon flesh grown in Brazil using ABTS radical scavenging activity at 222 ± 2 mg (ascorbic acid equivalent 100 g^−1^ DW) and its TPC at 275 ± 3 (mg gallic acid equivalent 100 g^−1^ DW) [19]. Yacon chips produced from yacon flesh grown in Bolivia were reported to have 9.7 ± 0.2 (mg gallic acid equivalent 100 g^−1^ FW) of TPC [20]. Therefore, the results of the previous investigations showed that yacon tubers and their processed food products contain considerable amounts of phenolic compounds and antioxidants, which can significantly vary according to cultivar, environmental conditions during cultivation, post-harvest, and processing conditions.

Similar to other fruits and vegetables, the availability of fresh yacon is seasonal [7]. Moreover, food processing such as drying, evaporation, and fermentation can be used to develop food products such as yacon chips, flour, syrup, vinegar, etc. to extend the shelf life of yacon tubers [7]. One of the major by-products of such food processing are the peels. Utilization of fruit peels as a source of valuable phyto/chemicals in nutraceuticals, value-added food products, pharmaceuticals, and cosmetic products has been introduced as an efficient and green strategy to reduce the waste in fruit production and consumption systems [21]. That being the case, the recovery of valuable nutritional compounds in the peels of various fruits has been suggested by several studies as they are considered to be a good source of phenolic and antioxidant compounds [22,23,24,25]. In respect to novel food product developments using yacon tubers, the flesh of tubers has been the focus of several recent studies, but yacon peels and recovery of their valuable compounds for potential applications has not been considered in detail yet [19,26,27,28]. A study of Pereira et al. (2016) investigated the phytochemical content in the peels and flesh of one yellow yacon cultivar cultivated in Brazil [29]. It was reported that these yacon peels had a TPC and ABTS radical scavenging activity of 2500.0 ± 23.1 (mg gallic acid equivalent kg^−1^) and 372.5 ± 15.9 (µmole trolox equivalent g^−1^ DW) [29]. Thus, differentiation between phyto/chemical content of flesh, peel, and whole yacon tuber is required to facilitate the selection of suitable raw material for specific food products to insure the aimed phyto/chemical quality of the final product.

Hence, the main objectives of this study were to evaluate the phytochemical content (TPC, total flavonoid content (TFC), ABTS radical scavenging activity, DPPH radical scavenging activity and FRAP in flesh, peel, and whole yacon tubers from seven cultivars—namely, Cajamarca, Cusco, Early White, Late Red, Morado, New Zealand, and Quinault grown under the same environmental conditions in Southwestern Germany. In addition, the sugar content (fructose, glucose, and sucrose) in the flesh, peel, and whole yacon tubers was investigated because it plays an important role in the sweetness of tubers and their resulting glycemic index.

## 2. Results and Discussion

### 2.1. Total Dry Matter Content

The statistical analysis of data indicated a significant interaction between cultivar and tuber part on total dry matter content of yacon tubers (*p* < 0.0001) (Table 1). The results for total dry matter content of different parts of yacon tubers are reported in Table 2.

The total dry matter content of flesh of yacon tubers ranged between 9.38 ± 0.40 to 15.13 ± 0.41 (g 100 g FW^−1^) and followed a decreasing order of cv. Morado > cv. Late Red > cv. New Zealand > cv. Early White > cv. Quinault > cv. Cusco > cv. Cajamarca (Table 2). Comparing the total dry matter content of whole tubers, the lowest values were determined for cv. Cajamarca (10.21 ± 0.41 (g 100 g^−1^ FW)) and cv. Cusco (10.17 ± 0.63 (g 100 g^−1^ FW)) while the highest significant values belonged to cv. Morado (16.93 ± 1.08 (g 100 g^−1^ FW)) (Table 3). Of the peels, the total dry matter content varied between 9.39 ± 0.84 (g 100 g^−1^ FW) for cv. Quinault and 15.68 ± 0.24 (g 100 g^−1^ FW) for cv. Morado (Table 2).

Total dry matter content of edible parts of plants is an important factor for determination of yield of crops as well as being a quality parameter which is related to the nutrient content of crops [30,31]. Total dry matter content in yacon tubers has been reported to range between 15 and 30 (g 100 g^−1^ FW) [32]. The results of total dry matter in this study were in agreement with previous investigations noting the total dry matter of 13.7 (g 100 g^−1^ FW) in yacon tubers cultivated in Japan [10], 7.5–19.1 (g 100 g^−1^ FW) in flesh of 35 accessions of yacon tubers cultivated in Peru [17], and 9.8–13.6 (g 100 g^−1^ FW) in yacon tubers of 10 accessions cultivated in Ecuador [33].

### 2.2. Glucose, Fructose and Sucrose Content

The statistical analysis of data showed that the interaction of cultivar and tuber part had a significant influence on glucose, fructose, and sucrose content in flesh, peel, and whole yacon tuber (*p* < 0.0001, *p* < 0.0001 and *p* < 0.0001, respectively) (Table 1). Table 3 reports the fructose, glucose, and sucrose content in different parts of yacon tubers.

The amount of fructose in the flesh of yacon tubers varied between 1.63 ± 0.30 (g 100 g^−1^ DW) for cv. Morado and 10.83 ± 0.37 (g 100 g^−1^ DW) for cv. Quinault (Table 3). The fructose content of whole tuber ranged between 0.17 ± 0.15 and 21.55 ± 0.74 (g 100 g^−1^ DW) for cv. Morado and cv. Quinault, respectively (Table 3). The peels of cv. Late Red and cv. Morado had the lowest amount of fructose at 0.04 ± 0.00 (g 100 g^−1^ DW) while the peels of cv. Quinault contained the highest fructose content of 3.09 ± 0.14 (g 100 g^−1^ DW) (Table 3). The glucose content of flesh of yacon tubers ranged between 0.18 ± 0.00 (g 100 g^−1^ DW) for cv. Late Red and 9.35 ± 0.36 (g 100 g^−1^ DW) for cv. Cajamarca (Table 3). The whole tuber of cv. Late Red and cv. Cajamarca contained the lowest and highest glucose content (0.29 ± 0.12 and 8.40 ± 0.25 (g 100 g^−1^ DW), respectively) (Table 3). The glucose content in peels varied between 0.17 ± 0.02 and 1.04 ± 0.06 (g 100 g^−1^ DW) for cv. Late Red and cv. New Zealand, respectively (Table 3). The results for sucrose content showed that the sucrose content in flesh of yacon tubers ranged between 21.00 ± 1.81 (g 100 g^−1^ DW) for cv. New Zealand and 53.54 ± 0.98 (g 100 g^−1^ DW) for cv. Early White (Table 3). The sucrose content in the whole tubers was lowest for cv. New Zealand (29.81 ± 0.47 (g 100 g^−1^ DW)). The highest sucrose content was found in the whole tubers of cv. Early White (58.08 ± 1.21 (g 100 g^−1^ DW)) (Table 3). The range of sucrose content in peels of yacon tubers was between 0.21 ± 1.26 and 29.89 ± 1.35 (g 100 g^−1^ DW) (for cv. Cusco and cv. Cajamarca, respectively) (Table 3).

Overall, the outcomes of examining the sugar content of flesh and whole tubers indicated that the amount of sugars in a decreasing order was as following: sucrose > fructose > glucose, with exception for cv. Cajamarca and cv. New Zealand for which the amount of sugars in a decreasing order was: sucrose > glucose > fructose (Table 3). The sugar content results for the peel showed that the sucrose content was higher than the fructose content while glucose content in the peel had the lowest amount in cv. Cajamarca, cv. Cusco, cv. Early White and cv. Quinault (Table 3). However, the order of sugar content in the peels of cv. Late Red, cv. Morado and cv. New Zealand was sucrose > glucose > fructose. Furthermore, in this study the sugar content of the yacon peel was reported for the first time. The outcomes showed that within each cultivar the sugar content of the peel was lower than that of the flesh and the whole tuber (Table 3). The low sugar content found in the yacon peel suggests its potential to be used in products with low sugar content.

A study of Graefe et al. (2004) reported sucrose, fructose, and glucose contents of 16.7–17.1, 10.5–15.2 and 1.1–3.3 (g 100 g^−1^ DW), respectively, in yacon tubers with different peel colors (purple, white, yellow) harvested in Peru [34]. The sugar content in the flesh of yacon tubers in their study had a decreasing order of: sucrose > fructose > glucose which is in agreement with the outcomes of this study [34]. A study of Lachmann et al., (2007) showed a different decreasing order of: fructose > glucose > sucrose content (19.5–21.7, 7.95–10.3 and 2.22–3.4 (g 100 g^−1^ DW) for yacon tubers from five different cultivars, cultivated in Czech Republic [35]. The fructose and glucose content of yacon tubers bought from a local market in Brazil were reported with 50.68 ± 0.1 and 26.93 ± 0.03 (g 100 g^−1^ DW), respectively [36]. A broader variation regarding the sum of reducing sugars and sucrose content (22.3–88.7 (g 100 g^−1^ DW)) in yacon tubers of thirty-five accessions cultivated in Peru was noted in a study of Campos et al. (2012) [17]. Fructose content and total sugar content (fructose + glucose + sucrose) of yacon tubers cultivated in Japan were reported at 35 and 58 (g 100 g^−1^ DW), respectively [10]. Such variations in sugar content may be due to the cultivar, environmental conditions during growth of yacon, and especially the post-harvest conditions as well as a possible combination of these factors [11]. In particular, the postharvest handling of yacon tubers contributes significantly to the simple sugar content and sweetness of tubers or products derived from them at consumption time [34]. For example, sunning of tubers is used as curing process for increasing the sweetness of tubers which causes the breakdown of FOS to FOS with lower degrees of polymerization and/or free fructose and glucose [34]. Food processes such as drying may also change the profile of carbohydrate content of yacon tubers [36]. As our tubers were not exposed to any post-harvest curing process, it is evident that no breakdown of FOS or sucrose had taken place yet leading to the determined order of sugars.

### 2.3. TPC

The statistical analysis showed that the amount of TPC in yacon tubers was significantly affected by an interaction of cultivar and tuber part (*p* < 0.0001) (Table 1). The amount of TPC in flesh, peel, and whole tubers of seven yacon cultivars is reported in Table 4.

Of the flesh, TPC of cv. Late Red was highest (3307.51 ± 21.84 mg GAE 100 g^−1^ DW) followed by cv. Cajamarca and cv. Morado containing 2710.39 ± 69.07 and 2571.95 ± 31.95 mg GAE 100 g^−1^ DW), respectively. The lowest TPC of 1855.89 ± 3.55 (mg GAE 100 g^−1^ DW) was measured in flesh of cv. New Zealand (Table 4). The TPC in whole tubers ranged between 2845.05 ± 0.87 and 3656.37 ± 74.60 (mg GAE 100 g^−1^ DW) for cv. New Zealand and cv. Morado (Table 4). A greater variation of TPC was found in the peels of yacon tubers from different cultivars when compared with the flesh and the whole tuber. TPC was lowest with 5602.23 ± 46.87 (mg GAE 100 g^−1^ DW) and highest with 14,144.53 ± 45.34 (mg GAE 100 g^−1^ DW) for cv. Late Red and cv. New Zealand, respectively (Table 4).

Variation in the TPC of tubers from different yacon cultivars was in agreement with variation in the TPC content of biomass [35] and flesh [17], as well as yacon flour [19]. Chlorogenic acid has been reported as a predominant phenolic compound in yacon roots while ferulic acid, coumaric acid, caffeic acid and its derivatives were identified in yacon root extracts [11,19,37]. Furthermore, the outcomes showed that the TPC was significantly higher in the peel of yacon tubers followed by the whole tuber and the flesh (Table 4). The effect of a cultivar on the TPC of plants or resulting food products as well as higher phenolic content in peels of other fruits and vegetables such as apples [23,38], bananas [25], potatos [24], and exotic fruits [22], has been reported by other researchers. Higher TPC in peel of fruits and vegetables might be in accordance to defense systems of plants. Phenolic compounds exhibit antioxidant and antimicrobial properties and their accumulation in the outer part of fruits and vegetables protects them against potential pathogens and harmful effects of the environment.

### 2.4. TFC

Results of ANOVA showed that the amount of TFC in the flesh, peel, and whole yacon tubers was significantly affected by the interaction of the cultivar and tuber part (*p* < 0.0001) (Table 1). Table 4 shows the TFC in the flesh, peel, and whole tuber of seven yacon cultivars.

The TFC of the flesh of yacon tubers varied between 1041.69 ± 25.36 and 4142.02 ± 471.981 (mg RE 100 g^−1^ DW) for cv. New Zealand and cv. Late Red, respectively, which is in agreement with results of TPC in the flesh being lowest and highest for the same cultivars (Table 4). Of the whole tubers, cv. Morado had the highest TFC of 4959.37 ± 340.51 (mg RE 100 g^−1^ DW), followed by similar values for cv. Cusco, cv. Late Red and cv. Early White at 4645.10 ± 126.29, 4365.77± 312.05 and 4147.11 ± 70.96 (mg RE 100 g^−1^ DW), respectively (Table 4). The lowest TFC in whole tubers was noted for cv. New Zealand (3221.47 ± 354.17 (mg RE 100 g^−1^ DW)) (Table 4). Similar to results of TPC, the peels of cv. New Zealand had the highest TFC (25,488.31± 554.02 (mg RE 100 g^−1^ DW)) and peels of cv. Late Red and cv. Morado were similar to each other according to the TFC. They contained the lowest TFC (9814.18 ± 1096.11 and 9670.46 ± 454.18 (mg RE 100 g^−1^ DW), respectively) (Table 4).

Flavonoids are polyphenolic compounds that contribute to sensorial properties of plant food products (e.g., taste and flavor). Further, they are considered to have antioxidant effects, anticancer activities, and antidiabetic effects [39]. To the best of our knowledge, this is the first study reporting the TFC of different parts of yacon tubers from various cultivars. Like TPC, the amount of flavonoids were influenced by cultivar which may suggest the influence of genotype on biosynthesis of flavonoid compounds in yacon plants. Of the flesh of yacon tubers, the amount of TFC was in the same range or higher than TPC in all cultivars except for cv. Cusco and cv. New Zealand. The latter two had respectively 9.28% and 43.87% lower TFC than TPC in the flesh of their tubers. The TFC in the flesh of cv. Late Red that had the highest TPC among investigated cultivars was 25.23% higher than its TPC. Moreover, for each cultivar the TFC, which were stored in the peel were higher than that of the whole tuber while the lowest TFC was measured in the flesh of tubers. This is in agreement with results of peels containing higher amounts of TPC (Table 4). Furthermore, these results are in agreement with results of TFC and their distribution among the peel and the flesh of apples [38,40], pears (peeled and unpeeled) [40], and potatoes [41]. In addition, it was noted that the TFC of whole tubers and peels was higher than their TPC content in all of the studied cultivars. More precisely, the TFC of whole tubers were higher than their TPC between 11.46% in case of cv. Cajamarca and 37.22% in case of cv. Late Red. Comparing the TFC and TPC in peel of tubers it was noted that TFC was profoundly higher than TPC within a range of 49.63% to 96.29% for peels of cv. Quinault and cv. Cajamarca, respectively. The results of previous studies regarding the individual flavonoids determined the presence of kaempherol, myricetin, quercetin and rutin in yacon leaves in various quantities depending on yacon cultivar, environmental conditions during cultivation, method of extraction and solvent used for extraction [42,43]. Simonovska et al. (2003) investigated the individual flavonoids in yacon leaves and tubers and suggested that yacon tubers might contain quercetin and other flavonoids, which remained unknown in their study [37]. Flavonoids are natural occurring antioxidants, which can be used to improve the health condition of consumers especially those who are suffering from diseases associated with oxidative stress such as diabetes [44]. Investigations showed a positive association between consumption of yacon tubers and improvement in health of diabetes, because of their hypoglycemic effect, which has been related to their FOS content [9]. The high TFC determined in this study might suggest that the consumption of yacon tubers might help to further improve the health conditions of consumers. Therefore, identification of individual flavonoid compounds in yacon tubers and their mechanism of action alone and in association with FOS, in vitro and in vivo are suggested.

### 2.5. Antioxidant Activity

#### 2.5.1. ABTS Radical Scavenging Activity

Statistical analysis of data showed that the interaction of the cultivar and tuber part had a significant effect on ABTS radical scavenging activity of yacon tubers (*p* < 0.0001) (Table 1). The ABTS radical scavenging activity of the flesh, peel, and whole tuber of seven yacon cultivars is presented in Table 5.

The ABTS radical scavenging activity of flesh was lowest for cv. Late Red at 366.81 ± 0.74 (mM TE 100 g^−1^ DW) and highest for cv. New Zealand at 407.62 ± 2.77 (mM TE 100 g^−1^ DW) (Table 5). Among the whole tuber of various cultivars, the lowest ABTS radical scavenging activity belonged to cv. Cusco (356.16 ± 0.52 (mM TE 100 g^−1^ DW)) while cv. New Zealand had the highest ABTS radical scavenging activity of 377.23 ± 0.43 (Table 5). The ABTS radical scavenging activity of peels was lowest and highest for cv. Morado and cv. Late Red at 261.98 ± 1.25 and 293.58 ± 0.98 (mM TE 100 g^−1^ DW), respectively (Table 5).

ABTS radical scavenging activity of the flesh, peel, and whole tubers was significantly affected by the cultivar (*p* < 0.0001) (Table 1). Furthermore, the results showed that the ABTS radical scavenging activity in the flesh of yacon tubers was higher than that of the whole tubers’. ABTS radical scavenging activity of the peels indicated the opposite trend compared to the results of TPC and TFC. Antioxidant activity of bioactive compounds and their mechanism of action with regard to free radicals are related to their structure. Therefore, the difference between TFC and TPC in different parts of the yacon tubers and their ABTS radical scavenging activity might be due to the structure of present phenolic and flavonoid compounds and their distribution in different parts of tubers [45,46]. ABTS radical scavenging activity in the flesh of yacon tubers of 35 yacon accessions was reported to range between 23 and 136 (µM TE 100 g^−1^ DW), which was lower than our findings [17]. Sousa et al. (2015) reported the ABTS radical scavenging activity of flour of flesh of yacon tubers with 222 ± 2 (mg ascorbic acid equivalent 100 g^−1^ DW) [19]. However, the results of the present study cannot be exactly compared to the results of the aforementioned studies, because of the differences in analytical methods for performing the measurement of ABTS radical scavenging activity. Furthermore, differences in solvents and extraction methods existed. Moreover, the environmental conditions during the cultivation may influence the phytochemical quality of plant food products leading to variations in their biological activity [47,48].

#### 2.5.2. DPPH Radical Scavenging Activity

Statistical analysis of data demonstrated that the DPPH radical scavenging activity of yacon tubers was significantly influenced by the interaction of the cultivar and tuber part (*p* < 0.0001) (Table 1). The outcomes of the DPPH radical scavenging activity of the flesh, peel, and whole tubers of seven yacon cultivars are presented in Table 5.

The DPPH radical scavenging activity of yacon tubers slightly varied when comparing different cultivars and tuber parts (Table 5). Of the flesh, the DPPH radical scavenging activity varied between 976.98 ± 103.18 and 1526.70 ± 2.22 (mg AAE 100 g^−1^ DW) for cv. New Zealand and cv. Morado, respectively. There was no significant difference between cv. Morado, cv. Late Red, cv. Quinault, cv. Cusco, cv. Cajamarca and cv. Early White according to their flesh’s DPPH radical scavenging activity (Table 5). The DPPH radical scavenging of whole tubers ranged between 1473.92 ± 16.20 and 1540.98 ± 13.76 (mg AAE 100 g^−1^ DW) for cv. Cajamarca and cv. Late Red (Table 5). The peels of yacon tubers had a DPPH radical scavenging activity between 1503.97 ± 10.55 and 1541.74 ± 7.97 for cv. Late Red and cv. Cusco (mg AAE 100 g^−1^ DW), respectively (Table 5).

Results of this study showed that no significant difference between DPPH radical scavenging activities of the different parts of yacon tubers of cv. Cajamarca, cv. Early White, cv. Late Red and cv. Quinault existed (Table 5). This might indicate an even distribution of antioxidant compounds in tubers, which can effectively scavenge free DPPH radicals in the peel and flesh of yacon tubers. However, the outcomes of DPPH radical scavenging activity are not in agreement with ABTS radical scavenging activity. The outcomes of the ABTS radical scavenging activity showed differences between the peel and flesh for all cultivars. The peel had a lower ABTS scavenging activity when compared to the flesh (Table 5). These results may indicate the variation in mechanism of action and effectiveness of bioactive compounds in yacon tubers against free radicals. The DPPH radical scavenging activity of yacon flesh has been studied by Yan et al. (1999) who separated and identified chlorogenic acid and l-tryptophan as two antioxidants in yacon tubers [49]. Additionally, it was determined that chlorogenic acid was much more effective than l-tryptophan in the scavenging of free DPPH radicals [49]. Moreover, Simonovska et al. (2003) noted the presence of an unknown non-polar compound which expressed high DPPH radical scavenging activity [37]. On that account, isolation and identification of bioactive compounds from the peel and flesh of yacon tubers can be suggested to determine the main antioxidant compounds and their mechanism action against oxidative stresses in vitro and in vivo.

#### 2.5.3. FRAP

The statistical analysis revealed a significant interaction between cultivar and tuber part on FRAP of yacon tubers (*p* < 0.0001) (Table 1). The results of FRAP of the flesh, peel, and whole tubers of seven yacon cultivars are noted in Table 5.

The FRAP of yacon tubers showed a great variation when comparing cultivar and different tuber parts. In the flesh of yacon tubers FRAP ranged between 6343.02 ± 74.17 and 24,393.48 ± 141.37 (mM Fe^2+^ 100 g^−1^ DW) for cv. New Zealand and cv. Late Red, respectively (Table 5). There was a significant difference between FRAP of whole tubers of all cultivars. The FRAP of whole tubers ranged between 17,020.23 ± 60.65 (mM Fe^2+^ 100 g^−1^ DW) for cv. Quinault and highest values of 25,418.22 ± 78.64 for cv. Morado (Table 5). The FRAP value of the peel was higher than that of the whole tubers and flesh and ranged between 27,959.12 ± 137.14 and 28,450.95± 35.15 (mM Fe^2+^ 100 g^−1^ DW) for cv. Morado and cv. Early White, respectively (Table 5).

To our knowledge, the present study is the first study that investigated the antioxidant activity of different parts of yacon tubers from various cultivars according to their reducing power. The findings showed that the ranking of tuber parts according to their FRAP values in a descending order is as follows: peel > whole tuber > flesh, except in the case of cv. Late Red which had the following decreasing order of: peel > flesh > whole tuber. This is consistent with the results of TPC and TFC (Table 4 and Table 5). Therefore, it might be suggested that flavonoid and phenolic compounds in different parts of yacon tubers are responsible for FRAP. Furthermore, higher FRAP values in the peel of yacon tubers was in agreement with higher FRAP in the peels of common fruits such as guava, white pomegranate, mango, kiwifruit etc. [50], as well as exotic fruits from Colombia such as coastal sapote, algarrobo, Borojo, and cassabanana [22].

### 2.6. Classification of Yacon Cultivars according to Their TPC and Total Sugar Content

Classification of various cultivars and breeding lines of plants is an important factor for breeding programs and food product development. For instance, potato tubers have been investigated in line with their phytochemical content in support of further breeding programs [51]. Moreover, potato tubers have been classified in compliance with their processing competence [52]. Such studies showed the importance of considering both nutritional factors and suitability of cultivars for specific food products with regard to final product characteristics for the selection of a cultivar for a certain purpose or for developing new cultivars.

**Figure 1:**
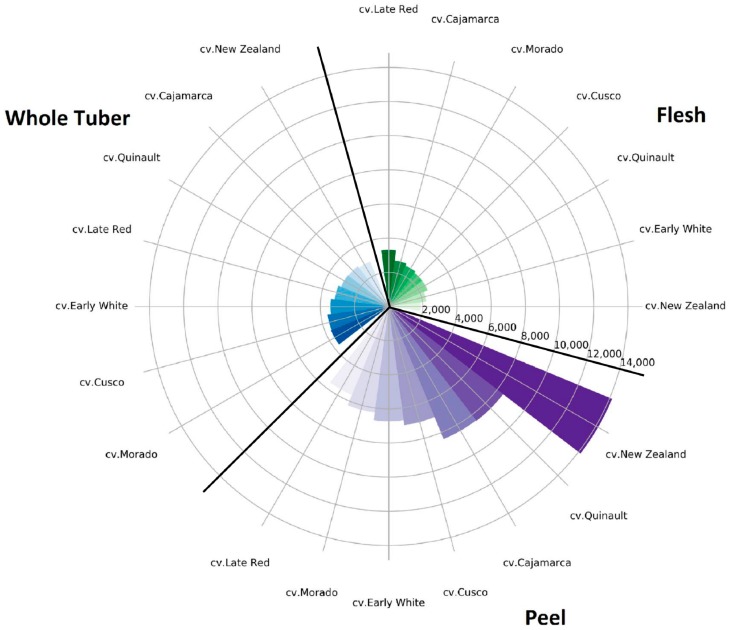
Classification of different parts (flesh, whole tuber, and peel) of the yacon tuber according to total phenolic content (mg GAE 100 g^−1^ DW).

**Figure 2:**
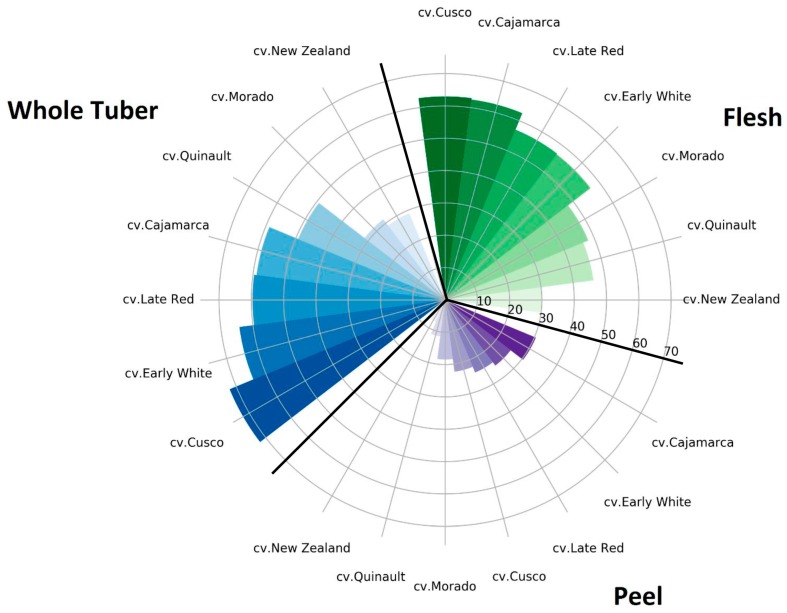
Classification of different parts (flesh, peel, and whole tuber) of the yacon tuber according to total sugar (fructose + glucose + sucrose) content (g 100 g^−1^ DW).

On the one hand, classification of yacon tubers as specified by their TPC can contribute to breeding programs that develop new cultivars containing beneficial nutritional traits. On the other hand, the sugar composition of yacon tubers is a deciding factor that determines their potential usage for development of trail-made food products for diabetics. In this regard, the cultivars with lower reducing sugar and sucrose content are more suitable as they lead to a lower glycemic index after consumption. Therefore, yacon cultivars might be classified in accordance to their sugar content or further selective breeding approaches to meet the specific dietary requirements of the target group of consumers, too. On that basis, the investigated yacon cultivars in the present study were categorized based on their TPC and total sugar content in the peel and whole tubers (Figure 1 and Figure 2).

As it is illustrated in Figure 1 and Figure 2, cv. Late Red and cv. Morado had the highest amounts of TPC in their flesh, while the total sugar content of their flesh was in a medium range. Consequently, they may be recommended for cultivation under European climatic conditions aiming for final food products with high health promoting compounds. The lowest total sugar content, TPC in flesh, and whole tuber was determined for cv. New Zealand while this cultivar also had the highest amounts of TPC and lowest amounts of total sugar in its peel (Figure 1 and Figure 2). That being the case, cultivation of cv. New Zealand under European environmental conditions might be of interest for the development of food products for diabetics.

## 3. Materials and Methods

### 3.1. Chemicals

Ascorbic acid, Folin–Ciocalteu’s reagent, FeCl_3_, FeSO_4_, HCl, NaNO_2_, NaOH, fructose, glucose, and sucrose were provided from Merck (Darmstadt, Germany). 2,4,6-Tris(2-pyridyl)-1,3,5-triazine (TPTZ) and 2,20-azino-bis(3-ethylbenzothiazoline-6-sulfonic acid) diammonium salt (ABTS), were purchased from Sigma (Darmstadt, Germany). AlCl_3_ (Fluka, Seelze, Germany). In addition, 2,2-diphenyl-1-picrylhydrazyl (DPPH) (CalBiochem, Darmstadt, Germany), Gallic acid (Scharlau, Barcelona, Spain), Na_2_CO_3_ (AppliChem, Darmstadt, Germany), potassium persulfate (Bernd Kraft, Duisburg, Germany), and Trolox (Cayman, Ann Arbor, MI, USA) were used. Methanol and ethanol were purchased from Chemsolute (Hamburg, Germany) and were HPLC grade.

### 3.2. Plant Material

Individual tubers from seven cultivars, which are presented in Table 6, were collected in October 2016 at harvest time from a field trial carried out at the research station Ihinger Hof of the University of Hohenheim (Stuttgart, Germany). Yacon rhizomes of all cultivars were purchased from Cultivariable (Moclips, WA, USA). Plantlets were cultivated in the greenhouse for 6 weeks and planted at the end of May 2016 in the field in hills (50 cm × 60 cm). The field was fertilized with 40 kg of nitrogen (ENTEC 26) before planting.

At harvest, tubers were washed with tab water and left in the open air to dry. Sample collection was done as follows: Tubers of one plant were cut in half and randomly divided into two portions. One portion was taken to be peeled manually with a hand peeler. Samples from flesh were collected by cutting flesh without peel into small cubic pieces (1×1×1 cm). Another portion of tubers was cut into small cubic pieces without being peeled and collected as a sample of a whole tuber. All samples were immediately frozen with liquid nitrogen and kept in a frozen state (−18 °C) before freeze drying. Afterwards, samples were freeze dried and milled.

### 3.3. Total Dry Matter Content

Total dry matter content of flesh, peel, and whole tuber samples was measured gravimetrically. The weight of samples was recorded before and after freeze drying and the total dry matter content was calculated using Equation (1).

(1) $$\mathrm{Total}\;\mathrm{dry}\;\mathrm{matter}\;\mathrm{content}=\frac{weight\;of\;samples\;after\;freeze\;drying}{weight\;of\;samples\;before\;freeze\;drying}\times 100$$

### 3.4. Determination of Glucose, Fructose, and Sucrose Content

Extraction of simple sugars was done according to the method used by Kolb et al. (2001) with slight modification [53]. Briefly, 0.1 g of sample powder was placed in a 250 mL Erlenmeyer flask and 50 mL ethanol (70%) was added to it. Then, the mixture was sonicated at 60 °C for 30 min. Afterwards, the mixture was allowed to cool down at room temperature. The extract was filtered using 0.45 µm nylon filters attached to a syringe.

High performance liquid chromatography (HPLC) was performed for determination of fructose, glucose, and sucrose content using a Dionex BioLC HPLC system (HPLC, Darmstadt, Germany). The device operated using a GS50 gradient pump, an AS 50 auto-sampler, an AS 50 Column oven, and DAD ED 50 Electrochemical Detector. Separation of sugars was done using Dionex CarboPac TM PA1 4 × 250 mm column and Dionex CarboPac PA1 40 mm pre-column at 25 °C. The mobile phase consisted of A (sodium hydroxide (150 mM)) and B (water) and C (sodium hydroxide (150 mM) + sodium acetate (500 mM)). It was eluted gradiently as follows for a total time of 20 min: 0 min (20% A + 80% B + 0% C); 10 min (20% A + 80% B + 0% C); 15 min (0% A + 0% B + 100% C); 18 min (0% A + 0% B + 100% C); and 20 min (0% A + 0% B + 100% C). An injection volume of 10.0 (μL) and a flow rate of 1 (mL/min) was applied. The fructose, glucose, and sucrose content in yacon extracts were determined using a standard curve drawn by injecting fructose, glucose, and sucrose (0–1 mg mL ^−1^).

### 3.5. Extraction of Phytochemicals

The extraction procedure was performed by adding 5 mL of methanol to 0.25 g of dried powder of yacon flesh, peel, and whole tuber. Then, the mixture was shaken (100 rpm) for 30 min at room temperature. Afterwards, the mixture was centrifuged (5810R, Eppendorf, Hamburg, Germany) at 4000 rpm for 10 min (20 °C) to separate the supernatant from the solid residuals. The methanol extracts were used for performing the following analysis:

#### 3.5.1. TPC

The TPC was determined following Folin–Ciocaltue methodology [54]. Briefly, 0.5 mL of prepared extract was mixed with 30 mL of distilled water in a 50 mL volumetric flask. After 6 min, 7.5 mL of sodium carbonate solution (20%) was added and the final volume was adjusted to 50 mL. The mixtures were left at room temperature for 2 h before reading the absorbance at 760 nm by means of a UV/Visible spectrophotometer (Ultrospec 3100 Pro, Amersham Bioscience, Buckinghamshire, UK). The standard curve was drawn using a gallic acid solution (0.3–3 mg gallic acid/mL distilled water) as a reference standard. TPC was expressed as gallic acid equivalent per 100 grams of dry weight (mg GAE 100 g^−1^ DW).

#### 3.5.2. TFC

TFC was measured as follows: 0.5 mL of extract was well mixed with 1 mL sodium nitrite solution (5%). After 6 min, 1 mL of AlCl_3_ (10%) and 10 mL of sodium hydroxide (1 M) was added to the mixture. The final volume of the mixture was adjusted to 25 mL by distilled water. Then, the mixture was kept for 15 min at room temperature. Finally, the absorbance was read at 510 nm using UV/Visible spectrophotometer (Ultrospec 3100 Pro, Amersham Bioscience). Rutin (0.0625–4 mg rutin/mL 70% ethanol) was prepared to generate the standard curve. TFC was expressed as rutin equivalent per 100 grams of dry weight (mg RE 100 g^−1^ DW) [55].

#### 3.5.3. Determination of Antioxidant Activity

##### ABTS (2,2′-Azino-bis(3-ethylbenzothiazoline-6-sulfonic Acid) Diammonium Salt) Radical Scavenging Activity

ABTS radical scavenging activity was measured following the method used by Dudonne et al. (2009) [56]. In order to produce ABTS radical cations (ABTS^+•^) potassium persulfate (2.45 mM) and ABTS solution (7 Mm) were mixed together and left to stand in the dark at room temperature for 12–16 h before use. The ABTS^+•^ solution was diluted to an absorbance of 0.700 ± 0.02 at 734 nm before being used. 3.0 mL of diluted ABTS^+•^ solution was added to 0.1 mL of extract. The reaction solution was maintained at 30 °C after mixing for 10 min. Then, the absorbance was read at 734 nm with UV/Visible spectrophotometer (Ultrospec 3100 Pro, Amersham Bioscience). The standard curve was generated using trolox solution (0.02–0.2 (mM)). ABTS radical scavenging activity was expressed as trolox equivalent per 100 grams of dry weight (mM TE 100 g^−1^ DW).

##### DPPH (2,2-Diphenyl-1-picrylhydrazyl) Radicals Scavenging Activity

The DPPH radical scavenging activity was measured as follows [56]: 0.1 mL of the extract was mixed to 3 mL of freshly prepared 6 × 10^−5^ mol/L DPPH^•^ solution in methanol. Afterwards, the reaction mixture was kept at 37 °C for 20 min before reading the absorbance at 515 nm using UV/Visible spectrophotometer (Ultrospec 3100 Pro, Amersham Bioscience). Ascorbic acid solution (0.02–0.2 mg ascorbic acid/mL distilled water) was used as a reference standard to draw the standard curve. DPPH radical scavenging activity was expressed as mg ascorbic acid equivalent per 100 grams of dry weight (mg AAE 100 g^−1^ DW).

##### FRAP

FRAP assay was performed as follows [57]: Fresh FRAP working solution was prepared by mixing 300 mM acetate buffer (pH 3.6), 10 mM TPTZ (2,4,6-Tris(2-pyridyl)-1,3,5-triazine) in HCl (10 mM) and 20 mM FeCl_3_ solution in a 10:1:1 (*v*/*v*/*v*) ratio. 0.15 mL of the extract was mixed with 2.85 mL of the FRAP solution and incubated at 37 °C for 30 min. The FRAP of the samples was evaluated by measuring the absorbance of Fe^2+^-TPTZ at 593 nm with UV/Visible spectrophotometer (Ultrospec 3100 Pro, Amersham Bioscience). The results of the FRAP assay were reported as Fe^2+^ (mM) equivalent per 100 grams of dry weight (mM Fe^2+^ 100 g^−1^ DW).

### 3.6. Statistical Analysis Of Data

Sample preparation and analysis were performed in duplicate and the results are reported as mean value ± standard deviation. For HPLC analysis, two extractions were performed for each sample and for each sample two injections were applied. The results were subjected to a two-way analysis of variance (ANOVA) (cultivar.tuber part) and the mean differences between evaluated parameters were established by performing Tukey’s test at 5% significance level. Statistical analysis of data was performed using SAS Software, version 9.4 (SAS Institute Inc., Cary, NC, USA). Figures were generated using matplot library from Python version 3.6.4 (Python Software Foundation, Wilmington, DE, USA).

## 4. Conclusions

The results of this study showed that the cultivar and yacon tuber part had a significant effect on the total dry matter content, sugars, TPC, TFC, and antioxidant activity of yacon tubers.

The ranking of the studied cultivars in decreasing order according to the total dry matter content of their flesh and whole tuber is as follows: cv. Morado > cv. Late Red > cv. New Zealand > cv. Early White > cv. Quinault > cv. Cusco > cv. Cajamarca. The total sugar content varied between cultivars. The lowest sugar content was noted for cv. New Zealand in the flesh, peel, and whole tuber. With regard to TPC, TFC, DPPH radical scavenging activity and FRAP of flesh and whole tubers, cv. Late Red, cv. Cajamarca, and cv. Morado were the three top cultivars while cv. New Zealand contained the lowest TPC and TFC when grown under European environmental conditions. However, the highest ABTS radical scavenging activity of the flesh and whole tubers was determined in cv. New Zealand and was the lowest for cv. Late Red which points to the importance of further investigations to determine the individual bioactive compounds.

Moreover, total dry matter content and phyto/chemical content of the peels of yacon tubers showed that the peels of yacon tubers are a good source of phytochemicals and exhibit considerable antioxidant activity while having low content of sugar. It was noted that the TPC, TFC and antioxidant activity of the peel of yacon tubers was higher than their flesh and even higher than those of whole tubers. The opposite trend was noticed for sugar content which was lowest in the peel of tubers. Therefore, it can be suggested that for minimizing the waste of food processing of yacon tubers the peels could use in other food and/or feed products, nutraceuticals, pharmaceuticals, and cosmetic products. However, more detailed investigations of the characterization of yacon peels is necessary to ensure their safety when being utilized as an ingredient in other products.

## Figures and Tables

**Table 1:** ANOVA results of total dry matter, fructose, glucose and sucrose content, total phenolic content, total flavonoid content, 2,20-azino-bis(3-ethylbenzothiazoline-6-sulfonic acid) (ABTS) radical scavenging activity, 2,2-diphenyl-1-picrylhydrazyl (DPPH) radical scavenging activity, and Ferric reducing antioxidant power as a function of the cultivar and different parts (flesh, peel, and whole tuber) of the yacon tubers.

	Total Dry Matter	Fructose Content	Glucose Content	Sucrose Content	Total Phenolic Content	Total Flavonoid Content	ABTS Radical Scavenging Activity	DPPH Radical Scavenging Activity	Ferric Reducing Antioxidant Power
Tuber part	*p* = 0.0003	*p* < 0.0001	*p* < 0.0001	*p* < 0.0001	*p* < 0.0001	*p* < 0.0001	*p* < 0.0001	*p* < 0.0001	*p* < 0.0001
Cultivar	*p* < 0.0001	*p* < 0.0001	*p* < 0.0001	*p* < 0.0001	*p* < 0.0001	*p* < 0.0001	*p* < 0.0001	*p* < 0.0001	*p* < 0.0001
Cultivar.Tuber part	*p* < 0.0001	*p* < 0.0001	*p* < 0.0001	*p* < 0.0001	*p* < 0.0001	*p* < 0.0001	*p* < 0.0001	*p* < 0.0001	*p* < 0.0001

**Table 2:** Total dry matter content (g 100 g^−1^ FW) of the flesh, peel, and whole tuber of different yacon cultivars.

Cultivar	Total Dry Matter Content (g 100 g^−1^ FW)		
	Flesh	Peel	Whole Tuber
Cajamarca	9.83 ^Db^ ± 0.40	11.77 ^BCa^ ± 0.11	10.21 ^Eb^ ± 0.41
Cusco	10.29 ^Db^ ± 0.32	12.51 ^Ba^ ± 0.44	10.17 ^Eb^ ± 0.63
Early White	12.77 ^BCa^ ± 0.62	10.20 ^DEc^ ± 0.12	11.67 ^Db^ ± 0.20
Late Red	14.13 ^ABa^ ± 0.62	12.58 ^Bb^ ± 0.67	14.69 ^Ba^ ± 0.27
Morado	15.13 ^Ab^ ± 0.41	15.68 ^Aab^ ± 0.24	16.93 ^Aa^ ± 1.08
New Zealand	12.94 ^BCa^ ± 0.61	10.80 ^CDb^ ± 0.26	13.30 ^Ca^ ± 0.64
Quinault	11.84 ^Ca^ ± 0.86	9.39 ^Eb^ ± 0.84	11.30 ^DEa^ ± 0.21

Reported values are presented as mean values ± standard deviation. Mean values with the same capital letter in a column are not significantly different as indicated by Tukey’s test (*p* < 0.05). Mean values with the same small letter in a row are not significantly different as indicated by Tukey’s test (*p* < 0.05).

**Table 3:** Fructose, glucose, and sucrose content (g 100 g^−1^ DW) in the flesh, peel, and whole tuber of different yacon cultivars.

Cultivar	Fructose (g 100 g^−1^ DW)			Glucose (g 100 g^−1^ DW)			Sucrose (g 100 g^−1^ DW)		
	Flesh	Peel	Whole Tuber	Flesh	Peel	Whole Tuber	Flesh	Peel	Whole Tuber
Cajamarca	8.54 ^Ba^ ± 0.42	0.37 ^Cc^ ± 0.13	7.35 ^Cb^ ± 0.24	9.35 ^Aa^ ± 0.36	0.18 ^Cc^ ± 0.00	8.40 ^Ab^ ±0.25	44.59 ^Ba^± 0.74	29.89 ^Ab^ ± 1.35	43.12 ^Ca^ ± 1.05
Cusco	10.64 ^Ab^ ± 0.42	0.50 ^Cc^ ± 0.13	16.99 ^Ba^ ± 0.60	6.65 ^Ca^ ± 0.29	0.47 ^Bb^ ± 0.02	6.88 ^Ba^ ± 0.25	45.62 ^Ba^± 0.98	21.00 ^Cb^ ± 1.26	48.31 ^Ba^ ± 3.10
Early White	2.14 ^Db^ ± 0.00	0.83 ^Bc^ ± 0.00	3.41 ^Ea^ ± 0.24	1.06 ^Eb^ ± 0.00	0.29 ^Cc^ ± 0.12	2.07 ^Da^± 0.12	53.54 ^Ab^± 0.98	24.45 ^Bc^ ± 0.00	58.74 ^Aa^ ± 2.01
Late Red	4.01 ^Ca^ ± 0.91	0.04 ^Dc^ ± 0.00	1.24 ^Fb^ ± 0.15	0.18 ^Fa^ ± 0.00	0.17 ^Ca^ ± 0.02	0.29 ^Ea^ ± 0.12	52.79 ^Ab^ ±3.92	24.09 ^Bc^ ± 0.42	58.08 ^Aa^ ± 1.21
Morado	1.63 ^Da^ ± 0.30	0.04 ^Db^ ± 0.00	0.17 ^Gb^ ± 0.15	0.51 ^Fa^ ± 0.12	0.24 ^Ca^ ± 0.00	0.45 ^Ea^ ± 0.00	45.85 ^Ba^ ± 0.84	18.26 ^Dc^ ± 0.87	31.00 ^Db^ ± 1.05
New Zealand	3.69 ^Cb^ ± 0.12	0.30 ^Cc^ ± 0.00	4.39 ^Da^ ± 0.15	5.33 ^Da^ ± 0.24	1.04 ^Ac^ ± 0.06	4.73 ^Cb^ ± 0.13	21.00 ^Da^ ± 1.81	6.00 ^Eb^ ± 0.77	19.81 ^Ea^ ± 0.47
Quinault	10.83 ^Ab^ ± 0.37	3.09 ^Ac^ ± 0.14	21.55 ^Aa^ ± 0.74	8.32 ^Ba^ ± 0.17	0.18 ^Cc^ ± 0.00	5.09 ^Cb^ ± 0.23	27.15 ^Ca^ ± 0.93	8.10 ^Fc^ ± 0.78	22.58 ^Eb^ ± 1.14

Reported values are presented as mean values ± standard deviation. Mean values with the same capital letter in a column are not significantly different as indicated by Tukey’s test (*p* < 0.05). Mean values with the same small letter in a row for each measured trait are not significantly different as indicated by Tukey’s test (*p* < 0.05).

**Table 4:** Total phenolic content (mg GAE 100 g^−1^ DW) and total flavonoid content (mg RE 100 g^−1^ DW) in the flesh, peel, and whole tuber of different yacon cultivars.

Cultivar	Total Phenolic Content (mg GAE 100 g^−1^ DW)			Total Flavonoid Content (mg RE 100 g^−1^ DW)		
	Flesh	Peel	Whole Tuber	Flesh	Peel	Whole Tuber
Cajamarca	2710.39 ^Bb^ ± 69.06	8402.94 ^Ba^ ± 221.45	2964.93 ^CDb^ ± 95.77	2726.80 ^Bb^ ± 120.81	16,494.91 ^Ba^ ± 324.01	3304.86 ^CDb^ ± 353.80
Cusco	2477.75 ^Cb^ ± 60.79	7007.29 ^Ca^ ± 475.86	3608.79 ^Ab^ ± 72.98	2247.73 ^BCc^ ± 221.44	12,959.57 ^Ca^ ± 214.57	4645.10 ^ABb^ ± 126.29
Early White	2213.73 ^Dc^ ± 38.59	6720.50 ^Ca^ ± 200.95	3397.55 ^ABb^ ± 61.64	2303.99 ^Bc^ ± 559.50	11,541.16 ^CDa^ ± 276.43	4147.11 ^ABCDb^ ± 70.96
Late Red	3307.51 ^Ab^ ± 21.84	5602.23 ^Da^ ± 46.87	3181.52 ^BCb^ ± 83.97	4142.02 ^Ab^ ± 471.98	9814.18 ^Da^ ± 1096.11	4365.77 ^ABCb^ ± 312.05
Morado	2571.95 ^BCc^ ± 31.95	6260.79 ^CDa^ ± 75.74	3656.37 ^Ab^ ± 74.60	2621.66 ^Bc^ ± 264.06	9670.46 ^Da^ ± 454.18	4959.37 ^Ab^ ± 340.51
New Zealand	1855.89 ^Ec^ ± 3.55	14,144.53 ^Aa^ ± 45.34	2845.05 ^Db^ ± 0.87	1041.69 ^Cc^ ± 25.36	25,488.31 ^Aa^ ± 554.02	3221.47 ^Db^ ± 354.17
Quinault	2470.21 ^Cc^ ± 9.29	8439.17 ^Ba^ ± 159.98	3005.57 ^CDb^ ± 83.59	2886.14 ^Bc^ ± 124.93	12,627.69 ^Ca^ ± 183.34	3593.64 ^BCDb^ ± 142.03

Reported values are presented as mean values ± standard deviation. Mean values with the same capital letter in a column are not significantly different as indicated by Tukey’s test (*p* < 0.05). Mean values with the same small letter in a row for each measured trait are not significantly different as indicated by Tukey’s test (*p* < 0.05).

**Table 5:** ABTS radical scavenging activity (mM TE 100 g^−1^ DW), DPPH radical scavenging activity (mg AAE 100 g^−1^ DW), and FRAP (mM Fe^2+^ 100 g^−1^ DW) of the flesh, peel, and whole tuber of different yacon cultivars.

Cultivar	ABTS Radicals Scavenging Activity (mM TE 100 g^−1^ DW)			DPPH Radical Scavenging Activity (mg AAE 100 g^−1^ DW)			FRAP (mM Fe^2+^ 100 g^−1^ DW)		
	Flesh	Peel	Whole Tuber	Flesh	Peel	Whole Tuber	Flesh	Peel	Whole Tuber
Cajamarca	376.40 ^Da^ ± 0.79	262.29 ^Cb^ ± 0.14	371.48 ^ABCa^ ± 3.50	1498.64 ^Aa^ ± 3.06	1513.45 ^Aa^ ± 9.88	1473.92 ^Ba^ ± 16.20	17,495.62 ^Bc^ ± 178.98	28,222.52 ^ABa^ ± 73.17	19,762.35 ^Eb^ ± 55.30
Cusco	384.81 ^Ca^ ± 0.13	266.19 ^Bc^ ± 0.40	356.16 ^Db^ ± 0.52	1498.85 ^Ab^ ± 3.88	1541.74 ^Aa^ ± 7.97	1507.47 ^ABb^ ± 0.12	15,521.66 ^Cc^ ± 59.38	28,450.95 ^Aa^ ± 35.15	24,508.18 ^Bb^± 184.62
Early White	397.30 ^Ba^ ± 1.84	264.68 ^BCc^ ± 0.04	369.43 ^BCb^ ± 1.27	1498.28 ^Aa^ ± 28.81	1509.07 ^Aa^ ± 3.47	1511.84 ^ABa^ ± 2.35	10,745.21 ^Ec^ ± 85.78	28,271.57 ^ABa^ ± 97.07	21,967.91 ^Db^ ± 92.47
Late Red	366.81 ^Eb^ ± 0.74	293.58 ^Ac^ ± 0.98	374.68 ^ABa^ ± 1.55	1520.51 ^Aa^ ± 8.54	1503.97 ^Aa^ ± 10.55	1540.98 ^Aa^ ± 13.76	24,393.48 ^Ab^ ± 141.37	28,040.22 ^Ba^ ± 116.32	22,852.36 ^Cc^ ± 34.32
Morado	392.25 ^Ba^ ± 1.27	261.98 ^Cc^ ± 1.25	372.28 ^ABCb^ ± 1.78	1526.70 ^Aab^ ± 2.22	1507.48 ^Ab^ ± 5.29	1540.91 ^Aa^ ± 11.34	15,137.52 ^Cc^ ± 19.02	27,959.12 ^Ba^ ± 137.14	25,418.22 ^Ab^ ± 78.64
New Zealand	407.62 ^Aa^ ± 2.77	262.18 ^Cc^ ± 1.82	377.23 ^Ab^ ± 0.43	976.98 ^Bb^ ± 103.18	1515.62 ^Aa^ ± 1.22	1513.12 ^ABa^ ± 1.96	6343.02 ^Fc^ ± 74.17	28,122.04 ^ABa^ ± 89.46	17,020.23 ^Gb^± 60.65
Quinault	393.00 ^Ba^ ± 2.53	263.34 ^BCc^ ± 0.03	367.05 ^Cb^ ± 0.83	1510.69 ^Aa^ ± 47.79	1507.32 ^Aa^ ± 25.72	1529.75 ^Aa^ ± 9.86	14,145.80 ^Dc^ ± 73.92	28,231.91 ^ABa^ ± 40.48	18,959.50 ^Fb^ ± 50.17

Reported values are presented as mean values ± standard deviation. Mean values with the same capital letter in a column are not significantly different as indicated by Tukey’s test (*p* < 0.05). Mean values with the same small letter in a row for each measured trait are not significantly different as indicated by Tukey’s test (*p* < 0.05).

**Table 6:** Color of the peel and the flesh of yacon tubers from different cultivars.

Cultivar	Peel Color	Flesh Color
Cajamarca	tan	white
Cusco	tan	white
Early White	tan	white
Late Red	red, tan	orange, yellow
Morado	purple	white
New Zealand	purple, tan	white
Quinault	white, tan	white
